# Cytokine and Chemokine Receptor Patterns of Human Malignant Melanoma Cell Lines

**DOI:** 10.3390/ijms23052644

**Published:** 2022-02-28

**Authors:** Viktoria Koroknai, István Szász, Krisztina Jámbor, Margit Balázs

**Affiliations:** 1 MTA-DE Public Health Research Group, University of Debrecen, 4032 Debrecen, Hungary; koroknai.viktoria@med.unideb.hu (V.K.); szasz.istvan@med.unideb.hu (I.S.); 2 Department of Public Health and Epidemiology, Faculty of Medicine, University of Debrecen, 4032 Debrecen, Hungary; jambor.krisztina@med.unideb.hu; 3 Doctoral School of Health Sciences, University of Debrecen, 4032 Debrecen, Hungary

**Keywords:** malignant melanoma, cytokine and chemokine receptor expression, invasion, BRAF mutation

## Abstract

Cytokine and chemokine receptors can promote tumor progression, invasion, and metastasis development by inducing different intracellular signaling pathways. The aim of this study was to determine the cytokine and chemokine receptor gene expression patterns in human melanoma cell lines. We found a large set of cytokine and chemokine receptor genes that were significantly differentially expressed between melanoma cell lines that originated from different subtypes of primary melanomas as well as cell lines that originated from melanoma metastases. The relative expressions of two receptor genes (*CCR2* and *TNFRSF11B*) were positively correlated with the invasive potential of the cell lines, whereas a negative correlation was observed for the *TNFRSF14* gene expression. We also found a small set of receptor genes that exhibited a significantly decreased expression in association with a *BRAF^V600E^* mutation. Based on our results, we assume that the analyzed cytokine and chemokine receptor collection may provide potential to distinguish the different subtypes of melanomas, helping us to understand the biological behavior of *BRAF^V600E^*-mutated melanoma cells.

## 1. Introduction

The signaling network between cells is essential for communication and regulation and can be facilitated by the interaction of cell surface receptors with secreted ligands [1]. Cytokines and chemotactic cytokines (chemokines) are soluble extracellular proteins that are integral parts of the signaling network among cells and can influence growth, development, hematopoiesis, lymphocyte recruitment, inflammation, and regulation of the immune system by binding to specific receptors [2]. Cytokine receptors can be divided into different groups, e.g., the hematopoietic receptor (HR) superfamily, the tumor necrosis factor (TNF) receptor superfamily, and interferon (IFN) receptors, based on their structural features [3]. The activation of cytokine receptors induces the Janus kinase/signal transducers and activators of the JAK/STAT transcription pathway [4]. Cytokines play a major role in different immune-related processes, and can also promote tumor growth and development [5].

Among the cytokines, chemokines are low-molecular-weight proteins that are able to regulate immune cell recruitment as well as tumor cell proliferation, invasiveness, and metastasis [6,7]. Their receptors are seven transmembrane domain G protein-coupled receptors (GPCRs), which are categorized based on their chemokine ligands (CC, CXC, CX3C, and XC families). Chemokine receptors can be activated by other types of chemokine ligands, whereas chemokines can bind to multiple receptors [2,6].

The role of chemokines and chemokine receptors in melanoma progression and metastasis formation has been described in several studies [8,9,10,11]. A melanoma is an aggressive, therapy-resistant malignancy that develops from melanocytes originating from a highly migratory embryonic cell population [12,13,14,15]. Metastasis formation is a multistep process that includes not only tumor cell invasion, in which cells have to pass through the basement membrane to enter the stroma, but also the arrest of tumor cells and extravasation into targeted organs, which involves cancer cell interactions with the host microenvironment [13,16].

Different in vitro and in vivo studies have shed light on the importance of the chemokine/chemokine receptor expression of the endothelial cells at the surface of the melanoma cells involved in organ-specific metastasis formations [17]. For instance, CCR6 expression has been described to associate with tumor cell migration and proliferation as well as tumor growth and lung metastasis formation in melanomas. CCR7 and CCR10 expressions have roles in regional lymph node metastasis, and CXCR4 is associated with the presence of ulceration, thicker lesions, migration, and invasion [17,18,19]. 

It is now established that cells from the primary tumor can prepare a metastatic niche before invasion. In the metastatic niche, immune cells and stromal cells—in collaboration with chemokines, matrix-degrading factors, and growth factors—stimulate the development of the metastatic lesion [20,21]. The aim of this study was to examine the cytokine and chemokine receptor expressions of various human melanoma cell lines and indicate the expression landscape of these receptors in context with the developmental stage, histological subtype, and invasive potential.

## 2. Results

### 2.1. Cytokine and Chemokine Receptor Expressions in Primary Tumors and Metastasis-Originated Melanoma Cell Lines

**Figure 1:**
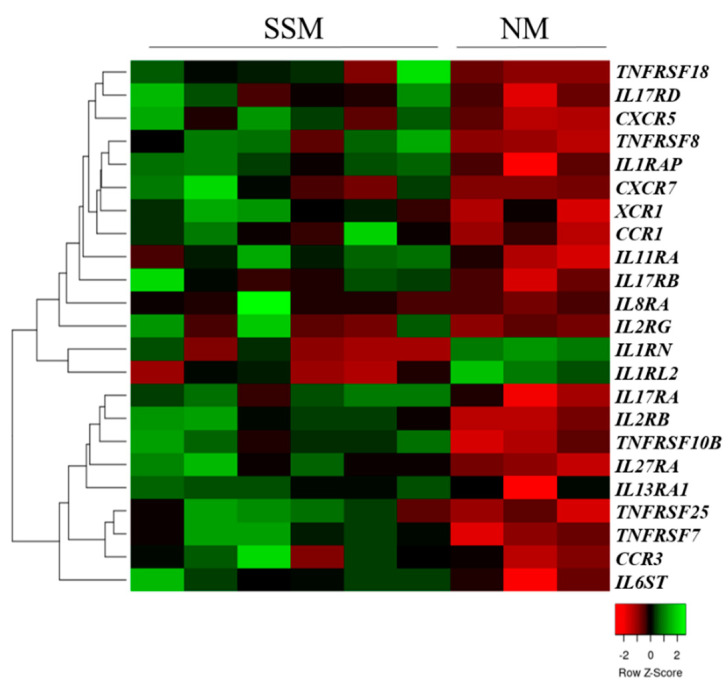
Unsupervised hierarchical clustering of cytokine and chemokine receptor genes that were differentially expressed in superficial spreading (SSM) and nodular (NM) melanoma-derived cell lines. The heat map was generated from the 23 significantly expressed genes (Mann–Whitney–Wilcoxon test, *p* < 0.05) using www.heatmapper.ca [22]. The cell lines are displayed vertically, and the genes are displayed horizontally.

Real-time qRT-PCR analyses were performed to examine the expression patterns of the cytokine (interleukin and tumor necrosis factor) and chemokine receptors of primary tumors (superficial spreading melanoma (SSM), *n* = 7; nodular melanoma (NM), *n* = 3) and metastasis-originated (MM, *n* = 9) melanoma cell lines. The expression of 13 interleukin receptor genes, five tumor necrosis factor receptor genes, and five chemokine receptor genes was significantly different between the SSM- and NM-originated primary melanoma cell lines (*p* < 0.05). Hierarchical clustering of the 23 differentially expressed genes clearly showed distinct expression between the NM and SSM subtypes of the originated cell lines (Figure 1). 

Most of the receptor genes were significantly downregulated in the cell lines that originated from the NM subtype. Only two genes (IL1RL2 and IL1RN) were significantly overexpressed in the NM-type cell lines compared with the SSM-derived cell lines (*p* < 0.05).

**Figure 2:**
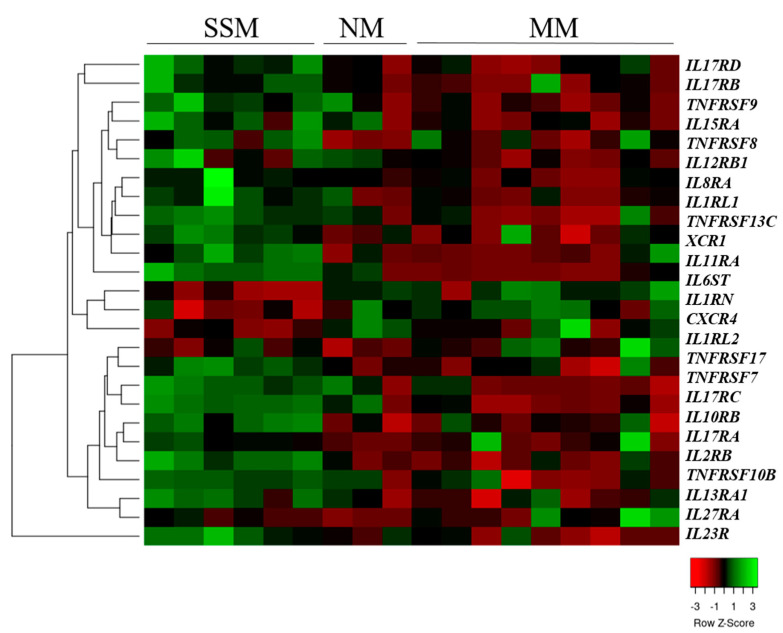
Unsupervised hierarchical clustering of cytokine and chemokine receptor genes differentially expressed in superficial spreading (SSM), nodular (NM) and metastatic melanoma-derived cell lines. The heat map was generated from the 26 significantly expressed genes (Kruskal–Wallis test, *p* < 0.05) using www.heatmapper.ca [22]. The cell lines are displayed vertically, and the genes are displayed horizontally.

We also compared the cytokine and chemokine receptor gene expressions of the SSM-, NM-, and MM-derived cell lines and found that two chemokine receptors, 17 interleukin receptor genes, and seven tumor necrosis factor receptor genes were significantly differentially expressed (Figure 2). 

From these 26 significantly altered genes, 14 were differentially expressed between the SSM- and NM-originated cell lines, whereas only two of the 26 (TNFRSF17 and IL27RA) significantly altered genes were found after comparing the NM- and MM-originated cells (*p* < 0.05). This indicated that the expression patterns of the cytokine and chemokine receptor genes in the NM-derived cell lines were more similar to the metastasis-originated cells than the SSM-originated cell lines. 

### 2.2. Cytokine and Chemokine Receptor Expressions in Association with a BRAF^V600E^ Mutation

**Figure 3:**
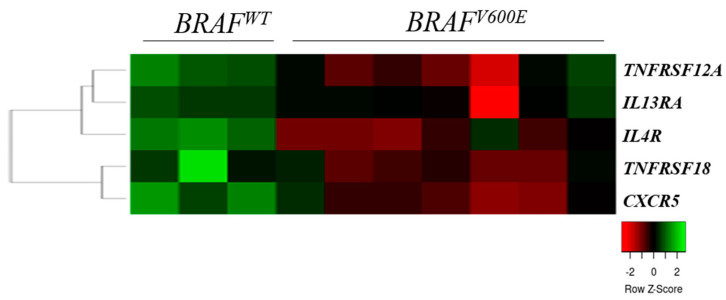
Heat map of the expression pattern of cytokine and chemokine receptors in wild-type (BRAF^WT^) and in mutant (BRAF^V600E^) primary melanoma-derived cell lines. The heat map was generated from the five significantly expressed genes (Mann–Whitney–Wilcoxon test, *p* < 0.05) using www.heatmapper.ca [22]. The cell lines are displayed vertically, and the genes are displayed horizontally.

We compared the cytokine and chemokine receptor gene expression data of seven BRAF^V600E^ mutant and three wild-type (BRAF^WT^) melanoma cell lines and found five receptor genes (TNFRSF12A, TNFRSF18, IL4R, IL13RA, and CXCR5) that were significantly overexpressed in the WT cell lines compared with the BRAF^V600E^ mutant cells (Figure 3).

### 2.3. Correlation of Invasive Behavior with Cytokine and Chemokine Receptor Expressions in Melanoma Cell Lines

To identify cytokine and chemokine receptor alterations in association with the invasive potential, we performed an in vitro invasion assay on 10 primary tumor-derived melanoma cell lines. The results are summarized in Table 1. Based on our data, five melanoma cell lines (WM793B, WM278, WM3211, WM1366, and WM983A) showed invasive behavior.

**Figure 4:**
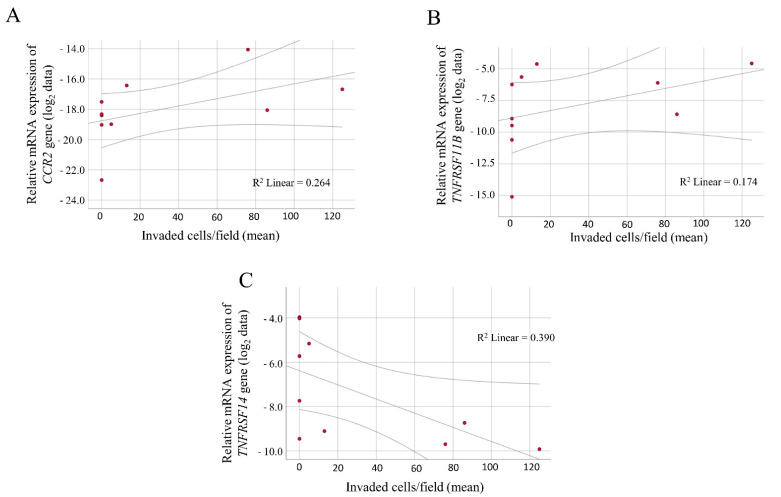
Correlation between the invasive behavior of primary melanoma-derived cell lines and the log_2_-transformed expression data. Positive correlations were found between the relative expression level of (**A**) the CCR2 chemokine receptor gene, (**B**) the TNFRSF11B tumor necrosis factor receptor gene and the number of invaded cells. (**C**) Negative correlation of the TNFRSF14 tumor necrosis factor receptor gene expression and invasion data. The qRT-PCR results are presented as the mean 2^−ΔCt^ values of two independent experiments. The results of the invasion assay are shown as the mean of three independent experiments.

Correlation analysis was performed between the number of invaded cells/field and the qRT-PCR expression data, which revealed a significant positive correlation between the relative mRNA levels of the CCR2 and TNFRSF11B genes (Figure 4A,B; R = 0.640, *p* = 0.046; and R = 0.743, *p* = 0.014, respectively). 

The relative expression level of the TNFRSF11B gene was significantly increased in the invasive cells compared with the non-invasive cells (*p* = 0.016). A significant negative correlation was observed between the relative expression of the TNFRSF14 gene and the invasive capacity of the melanoma cells (Figure 4C; R = −0.653, *p* = 0.041).

## 3. Discussion

Over the past years, a deeper knowledge of melanoma development and biology has been reached. However, the understanding of the whole mechanism of the phenotype switching to an increased invasion behavior and metastatic spread is still incomplete. As identified in the ‘seed and soil’ hypothesis, several organs can support the invasion, survival, and growth of tumor cells, but organ specificity observed in metastasis formation (organotropism) is one of the crucial unanswered questions in cancer research [23,24]. 

Recently, a large amount of evidence has accumulated on the important role of bioactive lipids/lipid signaling in the progression of malignant melanoma [25]. Sphingolipids, including two central bioactive lipids, sphingosine-1-phosphate (S1P) and ceramide, play a major role in melanoma progression, phenotypic changes, as well as regulation of chemokine and cytokine receptor expression [26,27]. Cytokines including interleukins, tumor necrosis factors, and chemokines have crucial roles in the tumor progression and metastasis formation of cells binding to their receptors and activating different signaling pathways [28,29]. However, the cellular response depends on the expression of specific receptors on the cancer cell surface [7,30]. In this study, we determined the cytokine and chemokine receptor gene expressions of 10 primary tumors and nine melanoma metastasis-originated cell lines to indicate the possible role of specific receptors in association with the clinicopathological parameters. 

The two most common subtypes of primary melanomas are superficial spreading melanoma (SSM) and the nodular melanoma (NM) [31]. Although SSM tissues are characterized by a slow horizontal growth, an NM is considered to have a rapid and vertically growing phase [32]. Recent studies suggest that SSMs and NMs may progress independently and not in a linear progression model, which highlights the importance of effective subtype-specific therapeutic strategies [33,34,35]. According to our results, SSM and NM cell lines were clearly separated by a significantly expressed set of cytokine and chemokine receptors. Metastatic melanoma (MM) tumor-derived cell lines were also involved in the comparison, revealing significantly altered chemokine receptor genes (*n* = 2), interleukin receptor genes (*n* = 17), and tumor necrosis factor receptor genes (*n* = 7). This group of receptors may therefore have potential to distinguish between the different subtypes of melanoma tissues. 

During metastasis formation, there is a switch from a proliferative to an invasive state through transcriptional reprogramming, which is a crucial event in melanoma cells. Verfaillie et al. described a gene expression-based signature to predict melanoma invasion [36]. Two genes, TNFRSF11B and TNFRSF14 that were correlated with the invasion capacity of melanoma cells in our experiments, are part of this prediction system. A good concordance was found with the expression data of Verfaillie et al., as a significantly increased expression of TNFRSF11B was characteristic in the invasive cells. Similar to the published data, the TNFRSF14 gene was downregulated in the invasive melanoma cells, which confirmed the negative correlation between TNFRSF14 and the invasion potential [36].

The expression of the CCR2 receptor gene was positively correlated with the invasive potential of melanoma cells. The role of CCR2 (activated by its ligands) in the induction of migration and invasion in tumor cells including melanomas has been reported in recent studies [37,38]. The therapeutic block of the CCL2/CCR2 axis has yielded promising results to slow the progression of several tumor types [39,40,41]; the therapy of melanoma-targeting CCR2 inhibited tumor growth in laboratory animals [42]. 

We also observed a small set of receptor genes where a decreased expression was significantly associated with a BRAF^V600E^ mutation. A BRAF mutation is the most frequent type of mutation in melanomas (40–50%) as well as a resulting aberrant MAPK pathway activation, cell cycle deregulation, and apoptosis inhibition [43,44]. The most promising treatments for melanoma patients are combinations of BRAF (vemurafenib, dabrafenib, encorafenib) and MEK inhibitors (cobimetinib, trametinib, binimetinib); however, acquired resistance to treatments develops in a large number of melanoma patients, which strengthens the significance of new therapeutic targets to improve patient survival [45,46,47]. Targeting chemokine or cytokine receptors appears to be controversial, as treatments also affect immune cells that are not specifically tumor cells. A combination of chemokine receptor blockers with other antagonists in melanoma patients could be a promising area for further investigations [17,48,49,50]. 

In conclusion, the expression of cytokine and chemokine receptors may provide new approaches to distinguishing different melanoma cells based on clinicopathological parameters or to predict the proliferative and invasive characteristics of melanoma cells.

## 4. Materials and Methods

### 4.1. Melanoma Cell Lines

Primary and metastatic tumor-derived melanoma cell lines were obtained from two different sources. Three cell lines (WM35, A2058 and M24) were purchased from the American Type Culture Collection (ATCC, Manassas, VA, USA), and 11 cell lines (WM793B, WM3211, WM1361, WM902B, WM39, WM278, WM983A, WM1366, WM3248, WM1617, and WM983B) were obtained from the Coriell Institute for Medical Research (Camden, NJ Jersey, USA). The WM165−1, WM266−4 and cell lines were obtained from Rockland Immunochemicals, Inc. (Limerick, PA, USA). The HT168, HT168-M1 were developed from the A2058 cell line [22]. The characteristics and the origins of the cell lines are summarized in Table 2. 

The cells were cultured in RPMI 1640 medium (Lonza Group Ltd., Basel, Switzerland) or MCDB153-L15 medium (Sigma-Aldrich Co. LLC, St. Louis, MO, USA) supplemented with 5–10% fetal bovine serum (Gibco, Carlsbad, CA, USA) at 37 °C in 5% CO_2_ according to the protocol of the supplier.

### 4.2. In Vitro Invasion Assay

The invasive potential of the primary tumor-derived melanoma cell lines was examined using BD Biocoat Matrigel invasion chambers (pore size: 8 μm, 24 wells; BD Biosciences, Bedford, MA, USA) as described before [52]. Briefly, the upper chamber was filled with 500 μL of the cell suspension in serum-free media (5 × 10^4^ cells/well) whilst a medium containing 10% FBS was applied to the lower chamber as a chemoattractant. After the cells were incubated for 24 h at 37 °C, the cells in the lower layer were fixed and stained. The invaded cells were counted using a light microscope in seven different visual fields at 200× magnification; the data are presented as the means ±SD of three independent experiments.

### 4.3. Real-Time Quantitative PCR Analysis

RNA was isolated using an RNeasy Plus Mini Kit (Qiagen GmbH, Hilden, Germany) according to the manufacturer’s protocol. The concentration and quality of the RNA were assessed using a NanoDrop and Bioanalyzer (Agilent Technologies, Palo Alto, CA, USA). RNA samples with a 260/280 ratio ≥ 1.8 were included in further analyses. Reverse transcription was performed on the total RNA (1000 ng) using a High Capacity cDNA Archive Kit (Applied Biosystems, Carlsbad, CA, USA) according to the manufacturer’s protocol. 

The relative expression level of 96 genes (20 chemokine receptor genes, 43 interleukin receptor genes, 25 tumor necrosis factor receptor genes, and eight housekeeping genes; Supplementary Table S1) was determined using a LightCycler^®^ 480 Real-Time PCR System (Roche Diagnostics, GmbH, Mannheim, Germany). The primer sets (Human Cytokine and Chemokine Receptor Primer Library) were provided by RealTime Primers (RealTimePrimers.com, Elkins Park, PA, USA). The data are presented as the mean of 2^−ΔCt^ values of two independent experiments.

### 4.4. Statistical Analysis

SPSS 19.0 (SPSS Inc., Chicago, IL, USA) was used for the statistical analyses. The Shapiro–Wilk test was used to evaluate the normality of the data. The Spearman’s correlation coefficient was calculated to correlate the qPCR data with the number of invasive cells. The Mann–Whitney–Wilcoxon test was used to compare the qPCR data, and the different clinicopathological parameters; *p* < 0.05 was considered to be statistically significant.

## Figures and Tables

**Table 1:** Invasive property of primary tumor originated melanoma cell lines.

Cell Line	Growth Phase ^a^	Histologic Subtype ^b^	Number of Invaded Cells/Field (Mean ± SD) ^c^
WM793B	RGP/VGP	SSM	125.5 ± 16.3
WM278	VGP	NM	86.0 ± 18.3
WM3211	RGP/VGP	SSM	76.5 ± 29.5
WM1366	VGP	n.a.	13.0 ± 1.4
WM983A	VGP	n.a.	4.6 ± 2.6
WM3248	VGP	n.a.	0.0 ± 0.0
WM35	RGP	SSM	0.0 ± 0.0
WM1361	VGP	SSM	0.0 ± 0.0
WM902B	VGP	SSM	0.0 ± 0.0
WM39	VGP	NM	0.0 ± 0.0

^a^ RGP: radial growth phase; VGP: vertical growth phase; ^b^ SSM: superficial spreading melanoma; NM: nodular melanoma; n.a.: not available; ^c^ data are presented as the mean ± SD of three independent invasion assay experiments.

**Table 2:** Characteristics of human melanoma cell lines.

**Primary Tumor Originated Melanoma Cell Lines ^a^**				
**Cell Line**	**Growth Phase ^b^**	**Histologic Subtype ^c^**	**BRAF Mutation Status ^d^**	**NRAS Mutation Status ^e^**
WM35	RGP	SSM	V600E	wt
WM793B	RGP/VGP	SSM	V600E	wt
WM3211	RGP/VGP	SSM	wt	wt
WM1361	VGP	SSM	wt	Q61L
WM902B	VGP	SSM	V600E	wt
WM39	VGP	NM	V600E	wt
WM278 ^p1^	VGP	NM	V600E	wt
WM983A ^p2^	VGP	n.a.	V600E	wt
WM1366	VGP	n.a.	wt	Q61L
WM3248	VGP	n.a.	V600E	wt
**Melanoma Metastasis Originated Cell Lines**				
**Cell Line**	**Location of** **Metastasis**	**BRAF Mutation STATUS**	**NRAS Mutation Status**	
WM1617 ^m1^	lymph node	V600E	wt	
WM983B ^m2^	lymph node	V600E	wt	
A2058	lymph node	V600E	wt	
HT168 ^×1^	originated from A2058	V600E	wt	
HT168-M1 ^×2^	originated from HT168	V600E	wt	
M24	lymph node	wt	Q61R	
WM165−1	lymph node	V600D	wt	
WM266−4	lymph node	V600D	wt	
WM239A	lymph node	V600D	wt	

^a^ tumor type of melanomas which the cell lines were derived from; ^b^ RGP: radial growth phase, VGP: vertical growth phase; ^c^ SSM: superficial spreading melanoma, NM: nodular melanoma, n.a.: data not available; ^d^ V: valine, E: glutamic acid, K: lysine, wt: wild-type; ^e^ Q: glutamine, L: leucine, R: arginine; ^p^ primary tumor derived cell line with metastatic pair from the same patient; ^m^ metastatic pair of primary derived cell line; ^×1^ HT168 cell line originated from the A2058 cells after subcutan injection in immunosuppressed mouse; ^×2^ HT168-M1 cell line originated from the HT168 cells after intrasplenic injection in immunosuppressed mouse [51].

## Data Availability

Not applicable.

## Supplementary Materials

The following supporting information can be downloaded at: https://www.mdpi.com/article/10.3390/ijms23052644/s1.
